# The Study of Rhabdomyolysis in the Elderly: An Epidemiological Study and Single Center Experience

**DOI:** 10.14336/AD.2017.0304

**Published:** 2018-02-01

**Authors:** Supakanya Wongrakpanich, Christos Kallis, Prithiv Prasad, Janani Rangaswami, Andrew Rosenzweig

**Affiliations:** Department of Medicine, Einstein Medical Center, Philadelphia, Pennsylvania, USA.; Department of Medicine, Einstein Medical Center, Philadelphia, Pennsylvania, USA.; Department of Medicine, Einstein Medical Center, Philadelphia, Pennsylvania, USA.; Department of Medicine, Einstein Medical Center, Philadelphia, Pennsylvania, USA.; Department of Medicine, Einstein Medical Center, Philadelphia, Pennsylvania, USA.

**Keywords:** rhabdomyolysis, elderly, immobilization, fall, polypharmarcy

## Abstract

Rhabdomyolysis is a syndrome caused by injury to skeletal muscle. There is limited data of rhabdomyolysis in the elderly. The objective of this study is to investigate demographic data, etiologies, laboratory values, prognostic factors, and mortality of rhabdomyolysis in the geriatric population. A 4-years retrospective chart review study was conducted. Our inclusion criteria were age above 65 years and creatinine kinase level excess five times of normal upper limit. Among 167 patients, 47.3% were male. The median age at diagnosis was 80.11 (66-101) years. The duration of follow up in the study ranged from 0 to 48 months. Fall (with or without immobilization) was the most frequent cause of rhabdomyolysis in 56.9%. The mean baseline glomerular filtration rate (GFR), GFR at diagnosis, and peak decline in GFR was 76.94, 48.96, and 54.41 cc/min respectively. The mean CK at diagnosis and peak CK was 5097.22 and 6320.07. There were 45 deaths (21%) over the span of 4 years. Multivariate analysis demonstrated that number of medications pre-admission (Meds No.), peak decline in GFR, and acute kidney injury (AKI) are independent predictors for overall survival for rhabdomyolysis in the elderly. To our knowledge, this is the first epidemiological study of rhabdomyolysis in the elderly. Falls (with and without immobilization) were the most common etiology. Meds No. (>8), peak decline in GFR (<30 cc/min), and evidence of AKI are associated with shorter overall survival and can serve as potential independent prognostic markers for rhabdomyolysis in elderly patients.

Rhabdomyolysis is a clinical condition caused by injury to skeletal muscle causing leakage of muscle-cell components into body circulation. The common clinical presentations are muscle pain and/or weakness, dark colored urine, and elevation of creatinine kinase (CK), usually more than five times of normal upper limit [1, 2]. There are multiple known causes of rhabdomyolysis in adults such as strenuous exercise, heat stroke, seizure, delirium tremens, hypokalemia, hypophosphatemia, enzyme deficiencies, trauma, burns, heat stroke myopathy, immobility, hyponatremia, hypernatremia, arterial emboli, endocrinopathies, prolonged immobilization, shock, infections, and medications [3-7].

Vigorous hydration is considered the hallmark for the treatment of this condition. The main purpose is to promote adequate diuresis, correct hypovolemia, and dilute the muscle toxic metabolites from muscle destruction [8-10]. The prognosis of rhabdomyolysis is considered good with overall survival estimated to be around 80% [11], but varies depend on underlying etiologies and complications [3, 8]. Complications of rhabdomyolysis include electrolyte imbalances, arrhythmias, acute kidney injury (AKI), acidosis, compartment syndrome, and disseminated intravascular coagulation [2]. Approximately 30-50% of patient with rhabdomyolysis will develop AKI [9, 12].

Since Grossman et al. published the early epidemiological data for rhabdomyolysis in 1974 [13], there have been multiple studies regarding rhabdomyolysis in adults[14-18]. In 2013, McMahon et al. developed a risk prediction score for mortality in adult rhabdomyolysis patients with age older than 18 years old. They found that age, female sex, cause of rhabdomyolysis, serum creatinine, CK, phosphate, calcium, and bicarbonate are independent prognostic factors of rhabdomyolysis [19].

In the elderly patients, rhabdomyolysis is a common problem, however, there is no dedicated study focusing on the epidemiology and prognosis. The objective of this study was to investigate demographic data, comorbidities, etiologies, incidence of acute kidney injury, laboratory values, prognostic factors, and mortality of rhabdomyolysis in the geriatric population.

## MATERIALS AND METHOD

### Subjects

All elderly patients (age above 65 at the time of diagnosis) who had been diagnosed with rhabdomyolysis (defined by serum creatinine kinase (CK) level excess five times of normal upper limit [1, 20]) and visited the Albert Einstein Medical Center as outpatients or inpatients between October 2012 and October 2016 were included in this study. All subjects with preexisting myositis, myopathy, muscular dystrophy, or and CK elevation considered due to acute myocardial infarction were excluded.

One hundred and seventy-one patients fulfilled the inclusion criteria. After 4 patients were excluded, data from 167 patients were analyzed.

### Study Design

We conducted a 4-year retrospective chart review to obtain the following data: age at diagnosis, gender, race, body mass index (BMI), follow-up duration, co-morbidities (Hypertension (HTN), diabetes mellitus (DM), congestive heart failure (CHF), coronary artery disease (CAD), Parkinson’s disease, dementia, ambulatory dysfunction, cerebrovascular accident (CVA), and chronic kidney disease (CKD)), number of medications pre-admission (Med No.), etiology of rhabdomyolysis, laboratory values, cause of death, and date of death. Overall survival (OS) was defined as the period between the date of diagnosis and the date of death from any cause.

Data was analyzed using SPSS statistics version 17.0. Demographic and descriptive characteristics were summarized using descriptive statistics. Pearson correlation was used to calculate the relationship between peak CK, peak Cr or peak decline in GFR. Univariate and multivariate analyses using Cox proportional hazards regression models were performed to evaluate the independent prognostic factors for rhabdomyolysis in the elderly. A two-sided P-value less than 0.05 was considered to be statistically significant. The study was approved by the Institutional Review Board (IRB) of Philadelphia.

## RESULTS

### Patient Characteristics

Among 167 elderly patients with rhabdomyolysis, 79 (47.3%) patients were male and 88 (52.7%) patients were female. African American was the majority race in this study (73.7%), followed by Caucasian (19.8%). The median age at diagnosis was 80.11 years, with an age range of 66 to 101 years. The duration of follow up in the study ranged from 0 to 48 months. The average BMI was 20.9 (8.88) kg/m^2^. The most common comorbidities were HTN (81.4%), followed by ambulatory dysfunction (46.7%), DM (32.3%), CKD (25.1%), dementia (21.6%), CAD (18.6%), CHF (17.4%), CVA (17.4%), and Parkinson’s disease (1.8%).

### Etiology of Rhabdomyolysis

Falls (with or without immobilization) was the most frequent cause of rhabdomyolysis in 56.9%, followed by unknown etiology in 12%, seizures in 5.4%, immobilization in 5.4%, sepsis in 4.8%, statins in 2.4%, trauma in 1.8%, post surgery in 1.8%, medication induced in 1.8%, DKA in 1.2%, neuroleptic malignant syndrome in 1.2%, alcohol intoxication in 1.2%, vigorous exercise in 0.6%, post cardiac arrest 0.6%, infection 0.6%, and limb ischemia in 0.6%.

### Medications and Laboratory Parameters

The mean number of medications on admission was 7.52, with the range of 0-21. The mean baseline creatinine, creatinine at diagnosis, and peak creatinine was 1.255, 2.562, and 2.876 mg/dL respectively. The mean baseline GFR, GFR at diagnosis, and peak decline in GFR were 76.94, 48.96, and 54.41 cc/min respectively. The mean CK at diagnosis and peak CK were 5097.22 and 6320.07.

**Table 1:** Clinical characteristics in rhabdomyolysis elderly patients between AKI and non-AKI groups.

Characteristics	Non-AKI (n=50)	AKI (n=115)	P values
Age	81.36(9.41)	79.57(9.05)	0.251*
Duration of follow up (months)	13.21(13.83)	12.26(12.99)	0.679*
Body mass index	21.94(8.89)	20.43(9.19)	0.728*
Number of medications pre-admission	6.98(4.48)	7.75(4.71)	0.336*
Cr baseline	1.33(1.46)	1.22(0.62)	0.631*
GFR baseline	83.6(39.80)	73.72(27.53)	0.085*
CK at diagnosis	3984.94(6652.55)	5643.71(6608.37)	0.146*
Cr at diagnosis	1.68(2.69)	2.90(2.91)	0.012*
GFR at diagnosis	75.60(37.68)	37.72(23.24)	<0.001*
CK peak	5837.22(10171.41)	6612.12(7155.41)	0.578*
Potassium on admission	4.32(0.80)	4.47(1.01)	0.363*
Phosphorus on admission	3.65(1.36)	4.37(1.22)	0.034*
Albumin on admission	3.01(0.76)	2.67(0.72)	0.025*
Cr peak	1.72(2.69)	3.34(3.19)	0.001*
Peak decline in GFR	81.72(43.99)	42.55(26.95)	<0.001*
CK discharge	1443.13(3367.84)	1531.25(4309.98)	0.900*
Cr discharge	1.23(1.25)	1.75(1.97)	0.091*
Race			
African American	37 (74%)	86 (74.78%)	0.582**
Caucasian	10 (20%)	22 (19.13%)	
Hispanic	1 (2%)	1 (0.87%)	
Asian	2 (4%)	2 (1.73%)	
Other	0 (0%)	4 (3.47%)	
Sex			
Male	20 (40%)	58 (50.43%)	0.217**
Female	30 (60%)	57 (49.56%)	
Death in the same admission	1 (2%)	15 (13.04%)	0.030**
HTN	39 (78%)	96 (83.48%)	0.337**
DM	12 (24%)	42 (36.52%)	0.107**
CHF	7 (14%)	22 (19.13%)	0.426**
CAD	6 (12%)	25 (21.74%)	0.141**
Parkinson’s disease	0 (0%)	3 (2.61%)	0.249**
Dementia	11 (22%)	25 (21.74%)	0.970**
Ambulatory dysfunction	21 (42%)	56 (48.69%)	0.428**
CVA	5 (10%)	24 (20.87%)	0.092**
CKD	9 (18%)	33 (28.70%)	0.287**
Cause of rhabdomyolysis			0.381**
Fall and/or immobilization	38 (76%)	64 (55.65%)	
Statin induced	0 (0%)	2 (1.73%)	
Accident/Trauma	1 (2%)	2 (1.73%)	
Unknkown	4 (8%)	16 (13.91%)	
Medication induced	0 (0%)	3 (2.61%)	
Surgery	1 (2%)	2 (1.73%)	
Burn	0 (0%)	1 (0.87%)	
Seizure	2 (4%)	7 (6.09%)	
Sepsis	0 (0%)	8 (6.95%)	
Diabetes ketoacidosis	0 (0%)	2 (1.73%)	
Neuroleptic Malignant Syndrome	1 (2%)	1 (0.87%)	
Vigorous exercise	1 (2%)	0 (0%)	
Alcohol	1 (2%)	1 (0.87%)	
Post cardiac arrest	0 (0%)	1 (0.87%)	
Lower extremity ischemia	1 (2%)	0 (0%)	
Infection	0 (0%)	1 (0.87%)	
Fall and statin induced	0 (0%)	2 (1.73%)	
Fall	24 (48%)	46 (0.40%)	0.339**

* Independent t-test

** Chi-square test Cr-Creatinine, GFR-glomerular filtration rate, CK-creatnine kinase, HTN-hypertension, DM-diabetes mellitus, CHF-congestive heart failure, CAD-coronary artery disease, CVA-cerebrovascular accident, CKD-chronic kidney disease

**Table 2:** Univariate analysis using Cox proportional hazard regression between overall survival and rhabdomyolysis variables.

Characteristics	Hazard Ratio	95% Confident interval	P value
Age	1.016	0.984-1.050	0.325
Sex	1.3.543694	0.900-3.190	0.102
BMI	1.016	0.737-1.400	0.925
Hypertension	0.454	0.199-1.031	0.590
Diabetes	1.216	0.577-2.561	0.607
Congestive heart failure	1.681	0.690-4.097	0.253
Coronary artery disease	0.954	0.408-2.230	0.913
Parkinson’s disease	3.543	0.431-29.127	0.239
Dementia	1.761	0.843-3.680	0.133
Ambulatory dysfunction	0.426	0.203-0.895	0.024
Cerebrovascular accident	1.580	0.678-3.682	0.289
Chronic kidney disease	1.620	0.825-3.180	0.161
Number of medications	0.947	0.882-1.017	0.135
Baseline Creatinine	0.948	0.075-11.980	0.967
Baseline GFR	0.960	0.896-1.028	0.247
CK at diagnosis	1.000	0.999-1.000	0.757
Creatinine at diagnosis	0.276	0.022-3.450	0.318
GFR at diagnosis	0.928	0.822-1.048	0.227
Peak CK	1.000	1.000-1.001	0.506
Serum Potassium at diagnosis	1.025	0.377-2.792	0.961
Serum Phosphorus at diagnosis	0.978	0.479-1.996	0.951
Serum troponin at diagnosis	1.000	0.999-1.000	0.287
Serum albumin at diagnosis	1.268	0.460-3.495	0.646
Peak creatinine	3.200	0.340-30.091	0.309
Peak GFR	1.096	0.960-1.253	0.176
CK at discharge	1.001	0.999-1.002	0.265
Creatinine at discharge	0.963	0.191-4.862	0.964
Acute kidney injury (Yes/No)	3.043	1.90-7.781	0.020
Need for dialysis (Yes/No/ESRD)	2.013	0.884-4.582	0.096

GFR-Glomerular filtration rate, ESRD-end stage renal disease

### AKI and Rhabdomyolysis

The incidence of AKI was 68.9%. We divided the patients into two groups according to the presence or absence of AKI during hospitalization. Significant associations were found between AKI and higher phosphorus on admission (4.37 vs 3.65, *P* 0.034), lower albumin on admission (2.67 vs 3.01, P0.025), and higher percentage of death during the same admission (13.04 vs 2.00%, P 0.030) (Table 1). In addition, it was expected that AKI group had higher Cr at diagnosis (2.90 vs 1.68, *P*=0.012), lower GFR at diagnosis (37.72 vs 75.60, *P*<0.001), higher Cr peak (3.34 vs 1.72, *P*=0.001), and lower peak decline in GFR (42.55 vs 81.72, *P*<0.001). There was no statistical difference between Cr at discharge or CK at discharge between two groups.

### Correlation between peak CK and peak Cr or peak decline in GFR

We found neither statistically significant correlation between peak CK and peak Cr (Pearson’s correlation coefficient 0.019, *P*=0.805), nor peak CK and peak decline in GFR (Pearson’s correlation coefficient 0.037, *P*=0.638).

### Prognostic factors for overall survival

There were 45 deaths (21%) over the span of 4 years. All cause mortality during the same hospitalization was 10.17%. The median survival of this study was 7 months.

Univariate analysis using Cox proportional hazard regression between overall survival and each variable was analyzed (Table 2). We allowed all variables with a univariate *p*-value less than 0.25 to enter into a multivariate analysis. Once in a multivariate analysis, a backwards selection procedure was used to remove variables with a p-value greater than 0.05. This generated three independent predictors of overall survival in this population: number of pre-admission medications (<8 vs ≥8) (Hazard Ratio (HR) 2.391, 95% Confident interval (CI) 1.166-4.902, GFR (<50 vs ≥ 50)(HR 2.039, 95%CI 1.004-4.141), and AKI (HR 3.326, 95% CI 1.139-9.715). Therefore, we found that Med No., AKI, and GFR are independent predictors for overall survival for rhabdomyolysis in elderly patients (Table 3).

**Table 3:** Multivariate analysis using backward selection model

Characteristics	Hazard Ratio	95% Confident interval	*P* value
Number of Medications (<8 vs ≥8)	2.391	1.166-4.902	0.017
Peak GFR (<30 vs ≥ 30 mg/dL)	2.039	1.004-4.141	0.049
Acute kidney injury	3.326	1.139-9.715	0.028

## DISCUSSION

In this study, we described for the first time the epidemiology and prognosis of rhabdomyolysis in the elderly. Falls (with or without immobilization) was the most frequent cause of rhabdomyolysis in the elderly in 56.9%, compared with previous studies in adult patients which revealed illicit drug use and alcohol [14-16, 21], infectious [15, 18], ischemic [17], and seizure [13] as the most common etiology of rhabdomyolysis.

Approximately 34% of elderly age above 65 years old experience at least one fall per year [22]. Fractures, decrease in functional status, and risk for institutionalization are known consequences of falls. Besides the physical consequences, falls can also lead to mental consequences, such as, fear of fall, depression, social isolation, and lack of confidence [23]. Further studies about fall prevention strategies would definitely be beneficial in terms of preventing rhabdomyolysis, fractures, deconditioning in the elderly and other mental consequences of falls in the elderly [24].

Polypharmarcy, the overuse of prescription medications more than medically necessary, is highly prevalent in elderly patients [25]. It leads to multiple negative consequences, including, falls, functional decline, cognitive impairments, and malnutrition states [25]. In this study, we found that average number of medications was 7.52, with the range of 0-21. More importantly, we discovered that the number of medications pre-admission (>8) was associated with shorter overall survival in elderly patients with rhabdomyolysis.

It has been approximated that AKI secondary to rhabdomyolysis is responsible for 7-10% of all cause AKI in the United States [10]. The incidence of AKI was 68.9% in our study. This incidence is higher compared to the previous study by Melli et al [21]. and Veenstra et al [17], who reported an incidence of rhabdomyolysis 46% and 51% respectively. One possible explanation of our higher incidence of AKI is the age difference. Our study included elderly patients age above 65 years old, compared with both previous studies, which included all adults and children age from 4 years [21] and 16 years [17]. There are known associations between AKI and aging, for example, older adults in critical care settings had 20% higher rate of developing AKI compared to younger adults [26].

We performed further analysis by categorizing patients into AKI and non-AKI group. We found that patients with AKI had significantly higher phosphorus levels and lower albumin levels on admission. These findings were consistent with the study by ward et al. who studied the profile of AKI in rhabdomyolysis in 157 participants. They found higher serum phosphorus level and lower serum albumin level in renal failure group compared with non-renal failure group (1.85 vs 1.06 mmol/L, P<0.001 and 30.8 vs 35.9 g/L, P=0.010) [27]. In addition, there were no significant differences between CK and Cr levels upon discharge between groups.

Statins responsible for approximately 2.4% cases of rhabdomyolysis in our study. The incidence is slightly lower than the 4.2% rate reported by Melli et al [21]. There are several well-established studies about statin-associated adverse muscular events. In 2015, Iwere et al. conducted a systematic review and meta-analysis about statins and myopathy in elderly patients[28]. They included 8 studies and found that statins increase the risk of rhabdomyolysis in the elderly with the odd ratio of 2.93 (95% CI 0.30-28.18). Based on our results, previous study by Melli et al [21], and the above-mentioned meta-analysis, statins increase the risk of rhabdomyolysis in the elderly compared to general population, however, the event is generally uncommon.

There are some limitations in our study. The first limitation is in the study’s nature. Due to the fact that this study is a retrospective chart review study, some data may be underestimated or underreported, such as, comorbidities and etiology of rhabdomyolysis. The second limitation is disparities in terms of population group. The study population was predominantly African American (73.7%) thus limiting extrapolation of this data across all populations. Lastly, we have limited sample size of 165 patients. Larger prospective studies in elderly patients would be crucial in elucidating the relationship between falls, geriatric syndromes, and rhabdomyolysis in the elderly.

## Conclusions

To our knowledge, this is the first epidemiological study of rhabdomyolysis in the elderly. We demonstrated that falls (with and without immobilization) were the most common etiologies for rhabdomyolysis in the elderly. Approximately two-third of patients developed AKI. The number of medications pre-admission (>8), peak decline in GFR (<30 cc/min), and the presence of AKI are associated with shorter overall survival and can serve as potential independent prognostic markers for rhabdomyolysis in elderly patients.
